# Loss of the Desmosomal Component Perp Impairs Wound Healing *In Vivo*

**DOI:** 10.1155/2010/759731

**Published:** 2010-06-23

**Authors:** Veronica G. Beaudry, Rebecca A. Ihrie, Suzanne B. R. Jacobs, Bichchau Nguyen, Navneeta Pathak, Eunice Park, Laura D. Attardi

**Affiliations:** ^1^Division of Radiation and Cancer Biology, Department of Radiation Oncology, Stanford University School of Medicine, Stanford, CA 94305, USA; ^2^Department of Genetics, Stanford University School of Medicine, Stanford, CA 94305, USA

## Abstract

Epithelial wound closure is a complex biological process that relies on the concerted action of activated keratinocytes and dermal fibroblasts to resurface and close the exposed wound. Modulation of cell-cell adhesion junctions is thought to facilitate cellular proliferation and migration of keratinocytes across the wound. In particular, desmosomes, adhesion complexes critical for maintaining epithelial integrity, are downregulated at the wound edge. It is unclear, however, how compromised desmosomal adhesion would affect wound reepithelialization, given the need for a delicate balance between downmodulating adhesive strength to permit changes in cellular morphology and maintaining adhesion to allow coordinated migration of keratinocyte sheets. Here, we explore the contribution of desmosomal adhesion to wound healing using mice deficient for the desmosomal component Perp. We find that *Perp* conditional knockout mice display delayed wound healing relative to controls. Furthermore, we determine that while loss of Perp compromises cell-cell adhesion, it does not impair keratinocyte proliferation and actually enhances keratinocyte migration in *in vitro* assays. Thus, Perp's role in promoting cell adhesion is essential for wound closure. Together, these studies suggest a role for desmosomal adhesion in efficient wound healing.

## 1. Introduction

A fundamental aspect of the skin's ability to maintain barrier function is its rapid response to wounding. Critical components of adult wound healing include the contraction of the dermal tissue beneath the wound, which helps draw the wound edges in proximity, and reepithelialization, in which keratinocytes become activated to proliferate and migrate as an invading sheet to resurface the wound [1–3]. Keratinocytes display a number of distinct characteristics during wound healing, including increased size, elongated polarized morphology, compromised cell-cell adhesion, and retracted keratin filaments [2, 4, 5]. In addition, they undergo enhanced proliferation several cell diameters away from the wound edge [1, 3, 6]. These changes are thought to reflect reprogramming of epidermal keratinocytes to ones dedicated to wound healing. 

The dynamic regulation of cell-cell adhesion junctions, including desmosomes, is thought to be an important facet of wound healing. Desmosomal adhesion junctions are initially destabilized at the wound front, presumably to facilitate proliferation and migration, and are reassembled later during the sealing of the epithelium [7, 8]. Desmosomes are multiprotein cell-cell adhesion complexes essential for maintaining the structural integrity of tissues through connection to the intermediate filament network [9–12]. Transmembrane desmosomal cadherins, known as Desmogleins and Desmocollins, mediate contact between apposing cells, and the cytoplasmic tails of these cadherins interact with Plakoglobin and Plakophilins, which connect to the intermediate filament cytoskeleton via Desmoplakin. Recently, the Perp tetraspan membrane protein was identified as an additional member of the desmosome [13]. Perp's critical role in desmosomal adhesion was evidenced by the presence of dramatic blisters observed in the epidermis and oral mucosa of mice lacking Perp as well as by the ultrastructural abnormalities observed in desmosomes of these mice.

The role of desmosomal adhesion junctions in wound reepithelialization has been queried through *in vitro* studies to examine the consequence of desmosome-deficiency on individual cellular functions critical for wound healing, such as proliferation, adhesion, and migration. Because of the complex nature of wound healing, however, it is unclear what effect desmosome dysfunction would have on proper wound repair *in vivo*. Loss of desmosomal components might result in enhanced keratinocyte proliferation and migration, which could facilitate wound closure. Indeed, desmosomal component loss enhances proliferation *in vivo* [13–15] and increases cell migration *in vitro* [16–19]. Furthermore, signaling cascades that drive cell proliferation and migration in response to wounding, such as EGFR signaling, induce desmosome dissolution, and therefore desmosome loss might promote wound healing by accelerating the effects of the factors activating these pathways [17, 20, 21]. Alternatively, desmosome loss might be expected to hinder wound healing, due to the lack of cell-cell junctions to enable efficient coordinated migration of epithelial sheets or the inability to seal the wound. However, the consequences of desmosomal protein deficiency in wound healing have not been addressed using an *in vivo* genetic model, in part due to the lethal phenotypes observed in many knockout mouse strains lacking individual desmosome components [22]. We circumvent this problem by using *Perp *conditional knockout mice that we generated to assess the role of desmosomes in epidermal wound healing *in vivo*. Our studies here reveal an important role for Perp in efficient wound closure.

## 2. Materials and Methods

All animal studies were approved by the Stanford University Administrative Panel on Laboratory Animal Care.

### 2.1. Wounding Study in Mice

Keratin 14CreER^T2^ mice were bred to Perp^fl/fl^ conditional knockout mice and kept on a 129/Sv; C57BL/6 mixed background [23]. At 6 weeks of age, 0.1 mg of tamoxifen diluted in corn oil was administered to mice for 5 consecutive days via intraperitoneal injection. 4 weeks later mice were anesthetized with avertin (2,2,2-Tribromoethanol, Sigma Chemical Corp. St. Louis, MO), shaved, and subjected to 6 mm punch biopsies using a Dermal Biopsy Punch (Miltex, York, PA). The area of the wounds was measured on a daily basis for 10 days. Paraffin sections from adult skin were processed by standard methods.

### 2.2. Immunofluorescence Analysis

Samples were deparaffinized, rehydrated, and unmasked using Trilogy (Cell Marque, Rocklin, CA) in a pressure cooker for 15 minutes according to manufacturer's instructions. Samples were then rinsed in PBS, blocked in PBS containing 5% normal goat serum (Sigma Chemical Corp.), 2.5% BSA (Sigma Chemical Corp.), and 0.01% Triton X-100 (Fisher Scientific, Pittsburgh, PA). Sections were incubated in primary antibody overnight at 4°C, rinsed in PBS with Tween-20 (0.01%), incubated with secondary antibody and DAPI for 1 hr at 37°C, washed in PBS, and then mounted in Mowiol (Calbiochem, San Diego, CA). Antibodies used in this study were directed against Perp (1 : 150; [13]), Dsg 1 (18D4; 1 : 100 Santa Cruz Biotechnology, Santa Cruz, CA), Desmoplakin (11-5F; 1 : 50; gift of David Garrod, University of Manchester, Manchester, UK) Dsg1/3 (32-2D 1 : 50; gift of David Garrod, University of Manchester, Manchester, UK), Loricrin (1 : 500; Covance, Princeton, NJ), PCNA (1 : 100; Santa Cruz Biotechnology), and Keratin 1 & 14 (1 : 500; Covance). Secondary antibodies were FITC or AlexaFluor594 conjugated donkey anti-rabbit or anti-mouse IgG (1 : 400; Jackson Immunoresearch, West Grove, PA, and Invitrogen, Carlsbad, CA, resp.). Fluorescence images were examined using a Leica DM6000B microscope (Leica Microsystems, Bannockburn, IL), and images were acquired using a Retiga Exi Camera (Q imaging, Surrey, British Columbia, Canada) and Image Pro 6.2 software from Media Cybernetics (Silver Spring, MD).

#### 2.2.1. Explant Assay

Skin explants from wild-type and *Perp^−/−^* newborns were grown as described [24]. A minimum of 8 biopsies per mouse per experiment was examined.

#### 2.2.2. Transwell Migration Assay

24 well transwell plates (Costar, Corning, NY) were coated for 1 hour with a collagen-fibronectin solution. Primary mouse keratinocytes were isolated as described [13]. 50 000 cells for each genotype were plated into each well in triplicate, and 24 hours later cells that had migrated through the transwell were fixed in 4% paraformaldehyde for 15', rinsed in PBS, and stained with 0.01% crystal violet. The number of migrated cells was counted in three separate 200× fields. The average number of cells migrated per well was averaged over three independent experiments.

#### 2.2.3. Adhesion Assay

Mechanical dissociation assays were performed as described [25] on mouse keratinocytes grown to confluence in 0.05 mM calcium media, then switched to 0.2 mM calcium for 24 hours. Cell fragments were counted in four different 100× fields, in triplicate, using a Leica M × 6 dissecting microscope (Leica Microsystems).

## 3. Results

### 3.1. Perp-Deficiency Delays Wound Healing In vivo

**Figure 1:**
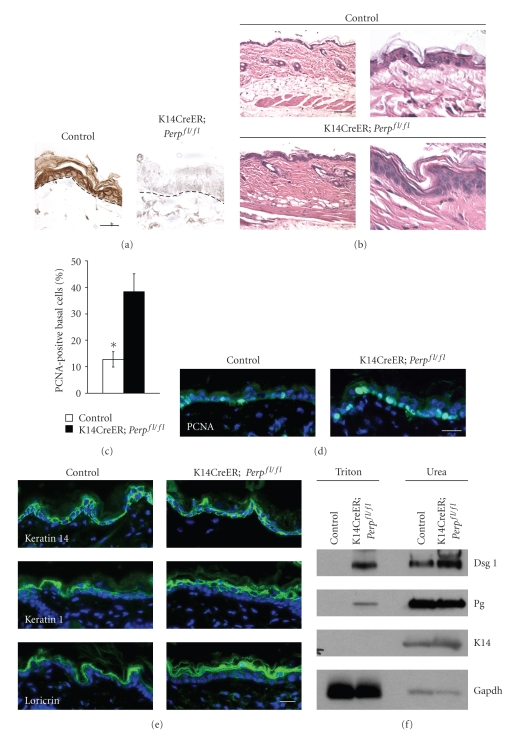
Acute deletion of Perp leads to desmosome defects. (a) Perp immunohistochemistry on skin samples from control (Perp^fl/fl^) and *K14CreER; *Perp^fl/fl^ mice 4 weeks post tamoxifen injection. (b) H&E analysis of control (Perp^fl/fl^) and *K14CreER; *Perp^fl/fl^ mice 4 weeks post tamoxifen injection, 200× and 400× magnification. (c) Graph displays the average percentage of PCNA positive cells in the basal cell layer in both control (Perp^fl/fl^) and *K14CreER; *Perp^fl/fl^ mice 4 weeks post tamoxifen injection. Graph represents the average of 3 separate fields from each of 4 mice +/− SEM. Statistical significance was determined using the Mann-Whitney test * = *P* < .03. (d) Representative image of the proliferation in the basal layer of the epidermis measured by PCNA immunostaining in control (Perp^fl/fl^) and *K14CreER; *Perp^fl/fl^ mice 4 weeks post tamoxifen injection. (e) Immunofluorescence analysis of differentiation markers on control (Perp^fl/fl^) and *K14CreER; *Perp^fl/fl^ mice 4 weeks post tamoxifen injection. Keratin 14, Keratin 1, and Loricrin mark the basal, the spinous, and the granular layers, respectively. DAPI is used to mark nuclei. (f) Solubility/western blot analysis of Dsg1 and Pg in *K14CreER; *Perp^fl/fl^ or control (*K14CreER; Perp^+/+^*) mouse skin. Both Triton X-100-soluble and Urea fractions are presented. Gapdh and Keratin 14 serve as loading controls for the Triton X-100 and urea fractions, respectively. Scale bar for panels (a, b) (right column), (d), and (e) equals 20 *μ*m. Scale bar for panel (b) (left column) equals  100 *μ*m.

To define the role of Perp and desmosomes in wound healing, we analyzed wound reepithelialization *in vivo* using Perp-deficient mice. We generated cohorts of 6-week-old control and conditional *Perp* knockout mice (Perp^fl/fl^; *fl* = floxed) expressing a *K14CreER* transgene, which allows deletion of the *Perp* locus in the epidermis upon introduction of tamoxifen. Immunohistochemistry confirmed that Perp expression was successfully abolished in the majority of these mice 4 weeks after tamoxifen injection (Figure 1(a)). Specifically, ~70% of these mice exhibited highly efficient Perp ablation (>90%) throughout the epidermis, while the other ~30% displayed somewhat less efficient Perp loss in the epidermis (>50%). Analysis of the skin from these mice revealed specific alterations in the epidermis of *K14CreER; *Perp^fl/fl^ mice compared to controls (Figures 1(b)–1(e)). For example, we noted an increase in the percentage of proliferating basal cells in the *K14CreER; *Perp^fl/fl^ mice compared to controls (Figures 1(c), 1(d)), similar to that observed in constitutive *Perp* knockout mice. Furthermore, this enhanced proliferation led to the expansion of differentiation marker staining in the skin (Figure 1(e)), although there were no apparent aberrations in the differentiation patterns of the skin in the *K14CreER; *Perp^fl/fl^ mice compared to controls. Occasional blisters were also observed in *K14CreER; *Perp^fl/fl^ mice, and accordingly, we found that desmosomes were functionally compromised in the skin of *K14CreER; *Perp^fl/fl^ mice using a solubility assay (Figure 1(f)). This assay relies on the fact that stably formed desmosomal complexes can only be solubilized by chaotropic agents, whereas improperly assembled desmosomal components can be solubilized by the nonionic detergent Triton X-100. We found that Desmoglein 1 and Plakoglobin display enhanced Triton X-100 solubility in skin from *K14CreER; *Perp^fl/fl^ mice compared to skin from control mice, confirming that acute deletion of Perp leads to impaired desmosome formation similar to that observed in constitutive Perp^−/−^ mice [13]. 

**Figure 2:**
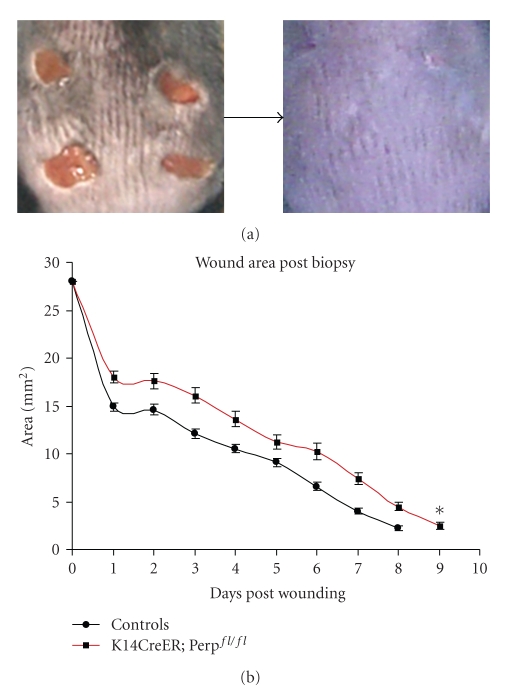
Loss of Perp delays wound healing *in vivo*. (a) Representative photos of dorsal skin bearing open wounds at the beginning of the study and fully closed wounds at the end of the study. (b) Graph displays the average area of all wounds as a function of time post-biopsy. Combined controls: *n* = 64 wounds amongst 16 mice. *K14CreER; *Perp^fl/fl^ mice: *n* = 32 wounds amongst 8 mice. Error bars represent the SEM. Statistical significance was determined using ANOVA * = *P* < .03.

To assay wound reepithelialization, we subjected mice in the Perp-deficient and control cohorts to full skin thickness punch biopsies. The experimental cohort consisted of tamoxifen-treated *K14CreER; *Perp^fl/fl^ mice, while the control cohorts comprised tamoxifen-treated *K14CreER; Perp^+/+^, *Perp^fl/+^, and Perp^fl/fl^ mice as well as untreated Perp^fl/fl^, Perp^fl/+^,* K14CreER; *Perp^fl/fl^, and *K14CreER; *Perp^fl/+^ mice. Four 6 mm punches were performed on each mouse, and the sizes of the wounds were measured daily for approximately 10 days to determine the rate of wound closure. We found that mice lacking Perp in the epithelial compartment exhibited a delay in wound healing relative to controls, underscoring the importance of Perp in the wound healing process (Figures 2(a), and 2(b)). 

**Figure 3:**
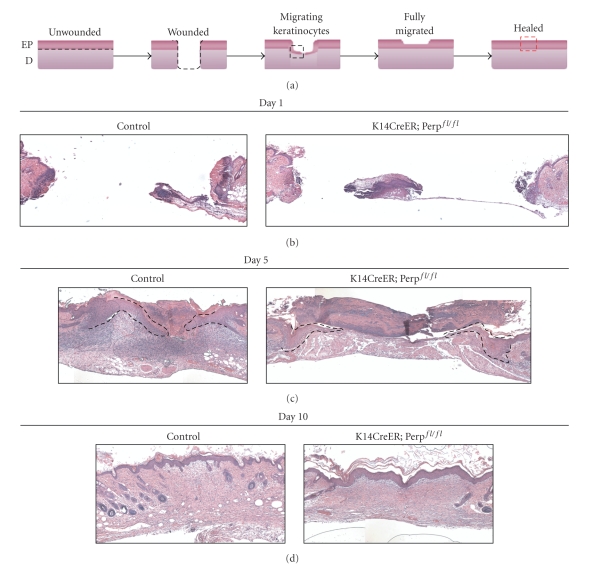
Wounded *K14CreER; *Perp^fl/fl^ mice exhibit delays in reepithelialization. (a) Cartoon sketch depicting the different stages occurring during the wound healing process. The black dashed line marks the boundary between the epidermis and dermis. Black dashed box demarcates region with migrating keratinocytes, as shown in panel (c). Red dashed box represents region of fully closed wound, as shown in panel (d). EP signifies epidermis and D, dermis. (b) Representative H&E-stained section of open wounds from tamoxifen-injected control (Perp^fl/fl^) and *K14CreER; *Perp^fl/fl^ mice 1 day post wounding. (c) Representative H&E-stained section of wounds from tamoxifen-injected control (Perp^fl/fl^) and *K14CreER; *Perp^fl/fl^ mice 5 days post wounding. The migrating epithelial sheets are outlined by the black dashed line. (d) Representative H&E-stained section of closed wounds from tamoxifen-injected control (Perp^fl/fl^) and *K14CreER; *Perp^fl/fl^ mice 10 days post wounding. Sizes of boxes in (b) and (c) were determined by the edges of the wound, as marked by the panniculus carnosus.

To further examine the wound healing process in the cohorts, histological analysis of transverse sections of wound sites from both control and *K14CreER; *Perp^fl/fl^ mice was performed 1, 5, 7, and 10 days post wounding (Figure 3, data not shown). These analyses revealed that while reepithelialization typically neared completion in the control mice by day 5, the epithelial cells had not fully migrated across wounds in the *K14CreER; *Perp^fl/fl^ mice (Figure 3(c)). In addition, by day 7, many of the wounds in control mice were not only fully reepithelialized but had reestablished normal epidermal architecture (data not shown). In contrast, in the *K14CreER; *Perp^fl/fl^ mice, keratinocytes had migrated across the wound but had not yet formed a full thickness skin layer by day 7 (data not shown). By day 10, both cohorts of mice exhibited fully reepithelialized wounds (Figure 3(d)). These data reinforce our observations at the macroscopic level that Perp loss delays wound healing after cutaneous injury.

### 3.2. Dynamic Changes in Desmosomal Protein Expression During Wound Healing in Wild-Type and Perp-Deficient Mice

**Figure 4:**
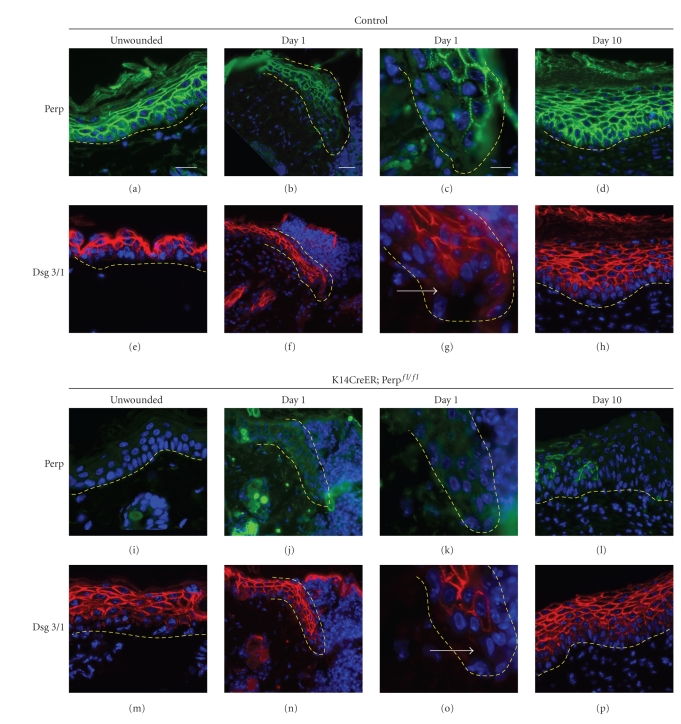
Migrating keratinocytes display reduced desmosomal protein expression at the wound edge. (a)–(d) Immunofluorescence images of Perp expression in the epithelium at different timepoints post wounding in control mice. The dashed yellow line represents the border between the migrating keratinocytes and underlying dermis. (e)–(h) Desmoglein 3/1 expression at different timepoints post wounding in control mice (tamoxifen-injected Perp^fl/fl^ mice). (i)–(l) Immunofluorescence images of Perp expression in the epithelium at different timepoints post wounding in *K14CreER; *Perp^fl/fl^ mice. (m)–(p) Desmoglein 3/1 expression at different timepoints post wounding in *K14CreER; *Perp^fl/fl^ mice. DAPI is used to mark nuclei. Arrows indicate cells with reduced desmosomal protein staining. Scale bar in (a) equals 20 *μ* *m*, scale bar in (b) equals 50 *μ* *m*, and scale bar in (c) equals 10 *μ* *m*.

The desmosomal component Desmoplakin has been reported to exhibit decreased expression in migrating keratinocytes at the leading edge of the wound both in keratinocyte scratch wound assays and in wounded epidermis [8]. However, adhesion remains intact between more distant cells of the epithelium, presumably to allow coordinated migration of the tissue. Examining changes in desmosomal protein localization during the stages of wound healing and determining how these patterns are affected in the absence of Perp may provide a clue to how Perp and potentially the desmosome are involved in wound closure. We examined the expression pattern of Perp and the desmosomal components Desmoglein 3/1 (Dsg3/1) and Desmoplakin (Dp) at different times after wounding *in vivo*, to determine their expression at the leading edge and in regions more internal to the wound. Analysis of samples from control mice 1 day post wounding revealed a decrease in membrane staining of Perp and Dsg3/1 near the leading edge of the epithelial tongue compared to uninjured epidermis (Figures 4(b), 4(c), 4(f) and 4(g)). By day 5, expression of Perp and Dsg3/1 became uniform throughout the migrating epithelium and by day 10, expression of all desmosomal components was restored to levels similar to those observed in uninjured epidermis (data not shown, and Figures 4(d) and 4(h)). Expression pattern changes for Dp were similar to those of other components (data not shown). These data support the notion that desmosome proteins are downregulated near the leading edge during the initial stages of the wound healing process but then are quickly restored, potentially to provide adhesive strength to the migrating epithelium. Similar patterns were observed in the wounds from *K14CreER; *Perp^fl/fl^ mice, suggesting that gross disruptions in desmosomal component targeting to the plasma membrane do not provide an explanation for the delayed wound closure observed in the Perp-deficient mice (Figures 4(i)–4(p)). We did note a few isolated migrating cells expressing Perp during the wound healing experiment, likely reflecting incomplete Perp deletion in stem cells of the interfollicular epidermis or the bulge. However, the number of cells expressing Perp was minimal and therefore unlikely to significantly affect our results. Importantly, any incomplete deletion we observed is likely to result in an underestimation of the extent of our phenotype.

### 3.3. Loss of Perp Does Not Enhance Proliferation or Apoptosis during Wound Reepithelialization

**Figure 5:**
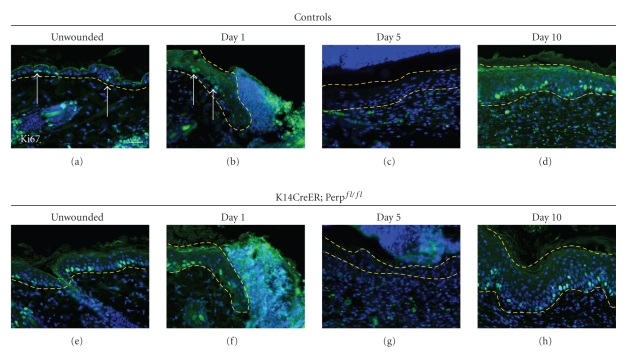
Migrating keratinocytes lacking Perp do not exhibit altered proliferation *in vivo*. (a)–(d) Representative images of Ki67 staining on sections of wounds at various timepoints post injury in control (Perp^fl/fl^) mice. (e)–(h) Representative images of Ki67 staining of wounds from *K14CreER; *Perp^fl/fl^ mice. Dashed yellow line surrounds epithelium. DAPI is used to stain nuclei. Arrows indicate Ki67 positive cells. Scale bar equals 50 *μ* *m*.

Defective wound closure could reflect alterations in keratinocyte proliferation. Specifically, enhanced cellular proliferation several cell diameters away from the edge is associated with wound reepithelialization [3]. We therefore examined whether the delayed wound healing observed in the absence of Perp was attributable to decreased cellular proliferation at and near the wound margin by staining for Ki67, a proliferation marker. Uninjured regions of control skin exhibit Ki67-positive cells scattered throughout the actively dividing basal layer (Figure 5(a)). One day post wounding, Ki67-positive cells were found in the area proximal to the wound edge (Figure 5(b)). However, at day 5, the epithelial sheet migrating across the wound expressed few Ki67-positive cells (Figure 5(c)). By day 10, Ki67-positive cells were observed throughout the basal layer of the healed epithelium, as in uninjured epidermis (Figure 5(d)). Upon analysis of Perp-deficient mice, we found no clear decrease in the levels of Ki67-positivity post wounding relative to controls (Figures 5(e)–5(h)). We also performed cleaved caspase 3 staining at days 1, 3, 5, and 10 post wounding to determine if there was any depletion of cells in the absence of Perp that could contribute to delayed wound healing, but we found no significant apoptosis in mice of either genotype (data not shown). Thus, altered levels of proliferation or apoptosis do not appear to account for delayed wound healing in Perp-deficient mice.

### 3.4. Perp Loss Disrupts Cell-Cell Adhesion and Enhances Keratinocyte Migration

**Figure 6:**
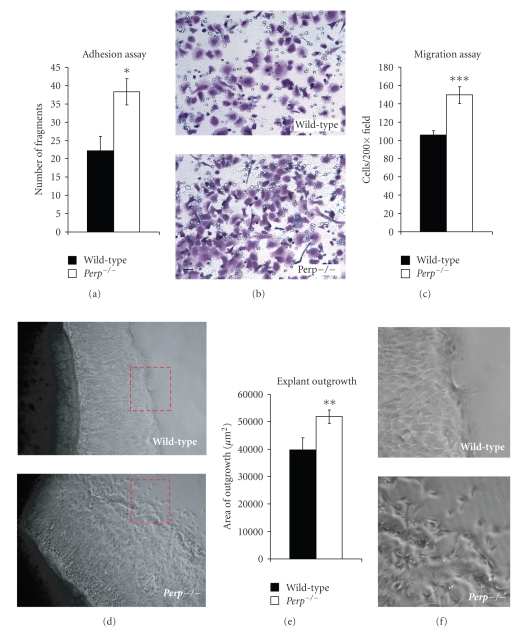
*P* *e* *r* *p* ^−/−^ keratinocytes display enhanced migration capacity *in vitro*. (a) A mechanical dissociation assay was performed on wild-type and *P* *e* *r* *p* ^−/−^ keratinocyte monolayers. The number of cellular fragments per 4 100× fields was counted and averaged. Graphs show the average of three independent experiments performed in triplicate and error bars represent +/− SEM. (b) A transwell migration assay was used to assess the migration potential of wild-type and *P* *e* *r* *p* ^−/−^ keratinocytes. Representative image of migrated cells stained with crystal violet. (c) The number of cells migrated in each of 3 200× fields was counted and averaged. The results represent the average of three individual experiments +/− SEM. (d) Representative phase-contrast images of wild-type and *P* *e* *r* *p* ^−/−^ explants. The red dashed boxes represent the area seen in (f). (e) The average areas of outgrowth after 10 days for wild-type and *P* *e* *r* *p* ^−/−^ explants are quantified in the graph. Wild-type: *n* = 26. *P* *e* *r* *p* ^−/−^: *n* = 50. Error bars represent the SEM. (f) Enlarged phase-contrast images of the areas boxed in red in panel (d) showing wild-type and *Perp* ^−/−^ explants. Statistical significance was determined using a Student's unpaired *t*-test * = *P* < .04,  ** = *P* < .03, *** = *P* < .003.

As defects in cell migration provide another potential mechanism for compromised wound healing, we next assessed effects of Perp loss on keratinocyte motility. First we confirmed that Perp loss leads to a demonstrable defect in cell-cell adhesion *in vitro*, where we planned to model migration, using a mechanical dissociation assay. This assay entails inducing desmosome formation in sheets of keratinocytes, detaching the epithelial sheet from the tissue culture dish using dispase, and incubating these sheets while rocking [25]. The number of fragments released provides a quantitative measurement of intercellular adhesive strength and the integrity of the epithelium. We found that Perp-deficient keratinocytes are indeed less adhesive than wild-type keratinocytes, as reflected by an increase in fragments seen after mechanical dissociation (Figure 6(a)).

We next used two different approaches to examine the role of Perp in regulating keratinocyte migration. We first examined cell migration using a transwell filter assay utilized previously to demonstrate that Plakoglobin-deficient cells display enhanced motility [26]. Wild-type and *Perp^−/−^* keratinocytes were grown in the upper chamber of transwell motility plates, and after 48 hours, their migration capacity was assessed by measuring the number of cells traveling through this filter to a lower chamber coated with collagen IV and fibronectin. Using this approach, we found that Perp loss does not compromise cell migration but instead augments it (Figures 6(b) and 6(c)). We also assessed migratory capacity in the context of an intact epithelial sheet using an *ex vivo* explant culture assay in which punch biopsies from mouse skin are cultured *in vitro* and the outgrowth of cells from the explant is measured, as a model for the behavior of cells at the edge of a wound [24]. We cultured punch biopsies of newborn skin from wild-type or constitutive *P* *e* *r* *p* ^−/−^ mice and monitored the outward migration of keratinocytes from the “wounded” edge of the biopsy over time. Consistent with the transwell assays, these experiments demonstrated that Perp-deficiency resulted in an enhanced area of outgrowth relative to wild-type mice, suggesting that cell migration is not defective, but instead is enhanced, in the absence of Perp (Figures 6(d) and 6(e)). Thus, a defect in migration *per se* does not account for the delayed wound healing observed in the Perp-deficient epidermis. Intriguingly, however, there appeared to be a qualitative difference in the way in which keratinocytes migrated in the absence of Perp. Specifically, despite migrating further, the leading edge of the migrating sheets from the *P* *e* *r* *p* ^−/−^ explants displayed a disorganized border, with cells detaching from one another and single cells transiting away. This phenotype is in contrast to that observed in the wild-type explants, in which keratinocytes appeared completely cohesive and moved as a coordinated epithelial sheet (Figure 6(f)). This disorganization, which is presumably a direct consequence of the impaired cell-cell adhesion seen in *Perp* null cells, could potentially contribute to the impaired wound healing *in vivo*, as coordinated migration is thought to be necessary for efficient wound reepithelialization.

## 4. Discussion

The regeneration of a functional epithelial barrier after wound healing is essential for preventing dehydration and infection [27]. Epithelial wound healing is a complex process, relying on the coordinated activation of fibroblasts mediating dermal contraction and keratinocytes responsible for reepithelialization of the wound as well as communication between these compartments [2, 3]. Differentiated keratinocytes undergo profound alterations to allow them to proliferate and migrate during the resurfacing of a wound. One notable change is the weakening of cell-cell adhesion at the leading edge of the wound. Although the dynamic regulation of cell-cell adhesion junctions like desmosomes has been suggested to be relevant for wound healing, the contribution of these complexes to efficient wound reepithelialization *in vivo* has not been elucidated genetically. Here, we queried the role of desmosomal adhesion junctions in epithelial wound closure using conditional knockout mice lacking the desmosomal component Perp. We observed a clear delay in wound healing in the absence of Perp, underscoring the importance of Perp and potentially desmosome function in epidermal cells for efficient wound closure *in vivo*. 

As wound healing relies on keratinocyte proliferation and migration, the delay in wound healing observed in the absence of Perp could reflect deficits in these processes. However, we found that keratinocytes lacking Perp do not display a defect in proliferation and, moreover, are more migratory than wild-type keratinocytes. This phenotype is consistent with other studies demonstrating that loss of desmosome components, including *PKP1, PKP3, Pg*, and *DP*, fails to affect proliferation but increases migration, either in scratch wound assays or in transwell assays [16, 18, 19, 26]. Instead of strictly affecting the rate of cell motility, the defective adhesion in *Perp* null cells could impede the ability of the migrating sheet to move efficiently in a coordinated manner, thereby potentially contributing to the delayed wound closure observed *in vivo*. This notion is consistent with the disorganized cells at the leading edge of the wound in the explant assays we performed. 

The ability of desmosomes to confer strength and resiliency to the skin relies on their connection to the keratin intermediate filament network. Analysis of mice deficient for keratins has supported the notion that intermediate filaments are important for efficient wound healing. Specifically, loss of Keratin 17 results in delayed wound healing using a mouse embryonic limb amputation system [28], and inactivation of its partner Keratin 6 impairs the closure of partial thickness wounds in adult mice [29]. These findings are consistent with the observation that induction of Keratins 6, 16, and 17 occurs rapidly in response to injury to the skin at the wound edge [28]. Given the tight linkage between the keratin intermediate filament network and the desmosome, it is possible that desmosomes would be affected in the keratin knockout mice and that their loss would contribute to the observed delay in wound healing [30, 31]. 

Mutations in genes encoding desmosomal components or the production of autoantibodies against desmosomal constituents have been associated with several human diseases. Mutations found in *PKP1, DSG1, PG,* or *DP* give rise to symptoms typical of ectodermal dysplasias, while autoantibodies against desmosomal proteins induce Pemphigus Vulgaris or Pemphigus Foliaceus, accompanied by blisters in the oral mucosa and/or skin [32, 33]. Patients bearing mutations in *PKP1* display chronic erosions and excessive scale-crust after trauma, suggesting a deficit in wound healing [18]. The inability of wounds to fully heal in these patients is likely attributable to defects in desmosome-mediated adhesion. Understanding how the desmosome participates in the wound healing process may lead to improved treatments for these patients. 

Our studies examining the role of Perp and desmosomes in epithelial wound healing have implications for other biological processes as well, as many of the events taking place during wound healing are important in other contexts. In particular, wound healing mimics the transitions occurring during a key developmental and tumorigenic process, epithelial-mesenchymal transition or EMT [34]. Consistent with this idea, Slug, a basic-helix-loop-helix transcriptional repressor required for certain characteristics of EMT, has been shown to be important for wound healing [35]. Slug is critical for wound outgrowth of cultured explants, and Slug overexpression induces desmosome dissolution, cell migration, and accelerated wound healing of keratinocytes [21, 35, 36]. Thus, studying the dynamics of desmosome behavior during wound healing may help illuminate pathways involved in development and cancer.

Our studies have highlighted a role for Perp in efficient wound closure. Not only is desmosome dissolution likely important at the leading edge of wounds early in wound healing, but intact desmosomal function may also be important for proper wound closure, potentially for efficient migration of the epithelium. These data suggest that precise spatial and temporal regulation of desmosomal complexes may be critical for wound healing. Future investigation into the signals regulating desmosome dynamics will provide further insight into both wound healing and cancer.
